# Long term effect of structured aerobic training versus structured resistance training program on glycaemic control in type 2 diabetes mellitus

**DOI:** 10.6026/973206300223248

**Published:** 2026-06-30

**Authors:** Kiran Mendhe, Praveen Kumar, Pallavi Kadve

**Affiliations:** 1 Department of Musculoskeletal Physiotherapy, VSPM's College of Physiotherapy, Lata Mangeshkar Hospital, Digdoh Hills, Hingana, Nagpur, India; 2 Department of Cardiovascular Physiotherapy, VSPM's College of Physiotherapy, Lata Mangeshkar Hospital, Digdoh Hills, Hingana, Nagpur, India; 3 Department of Biochemistry, NKP Salve Institute of Medical Sciences and Research Centre &Lata Mangeshkar Hospital, Digdoh Hills, Hingana, Nagpur, India

**Keywords:** Aerobic exercise (AE), lipid profile, resistance training, type 2 diabetes mellitus (T2DM), exercise therapy

## Abstract

The absence of clear evidence regarding the long-term comparative effectiveness of structured aerobic versus resistance training in improving lipid profile and metabolic parameters in type 2 diabetes mellitus remains a significant clinical challenge. Hence, this randomized controlled trial evaluated 60 patients with T2DM assigned to aerobic or resistance training groups over an extended follow-up period. Both interventions demonstrated improvements in glycaemic control, lipid profile, cardiovascular parameters and behavioral outcomes, with resistance training showing superior early-phase benefits in diet-related behaviors and metabolic adaptation. However, long-term outcomes between the two groups converged, indicating comparable efficacy over time. Thus, date shows that both exercise modalities are beneficial, with modality-specific temporal advantages. By providing controlled longitudinal evidence, this study advances knowledge by clarifying the time-dependent differential impacts of aerobic and resistance training on metabolic and behavioral outcomes in T2DM.

## Background:

Type 2 diabetes mellitus (T2DM), a chronic metabolic disorder characterized by persistent hyperglycaemia due to insulin resistance, impaired insulin secretion, or both. T2DM is strongly associated with multiple metabolic abnormalities, including dyslipidaemia, hypertension and obesity, all of which contribute significantly to cardiovascular morbidity and mortality [1]. As a result, comprehensive management strategies are essential, extending beyond pharmacological therapy to include lifestyle modifications such as diet and physical activity. Among these, exercise has emerged as a cornerstone intervention in the prevention and management of diabetes and its complications [2]. Aerobic exercise (AE) and resistance exercise (RE) are the two principal forms of physical activity recommended in clinical practice. Aerobic exercise primarily improves cardiovascular endurance and enhances insulin sensitivity through increased glucose uptake in skeletal muscles [3]. Resistance exercise, on the other hand, promotes muscle hypertrophy, increases basal metabolic rate and improves glucose utilization by augmenting muscle mass. Despite extensive research supporting the benefits of both exercise modalities, there remains a lack of consensus regarding their comparative effectiveness, particularly in long-term interventions [4]. Previous studies have demonstrated that regular exercise improves glycaemic parameters such as glycated haemoglobin (HbA1c), fasting blood glucose (FBG) and post-prandial blood glucose (PPBG) [5]. However, findings have been inconsistent regarding whether aerobic or resistance training is superior. Some studies suggest that aerobic exercise is more effective in improving post-prandial glucose and cardiovascular fitness, while others report greater benefits of resistance training in enhancing insulin sensitivity and lipid metabolism. These discrepancies may be attributed to variations in study design, duration, sample characteristics and adherence to exercise protocols [6]. In addition to glycaemic control, T2DM is frequently associated with dyslipidaemia; characterized by elevated low-density lipoprotein (LDL), increased triglycerides and reduced high-density lipoprotein (HDL). Exercise interventions have been shown to improve lipid profiles; however, evidence suggests that resistance training may have a more pronounced effect on HDL and LDL levels, whereas aerobic exercise may be more effective in reducing body fat and improving cardiovascular endurance. The need to clarify these differential effects is clinically important for optimizing exercise prescriptions [7]. Furthermore, hypertension is a common comorbidity in individuals with T2DM, further increasing the risk of cardiovascular complications. Both AE and RE have been shown to reduce systolic and diastolic blood pressure, but their relative effectiveness remains debated. Long-term studies evaluating these outcomes are limited, particularly in controlled clinical settings [8]. Therefore, it is of important to addresses these gaps by conducting a randomized controlled trial comparing the long-term effects of structured aerobic and resistance training programs on glycaemic parameters, lipid profile and cardiovascular outcomes in patients with T2DM.

## Methodology:

A total of 60 participants were included in the study and were randomly divided into two equal groups, with 30 participants assigned to the aerobic exercise group and 30 participants assigned to the resistance exercise group. The eligibility criteria included patients aged 30 to 60 years who had type 2 diabetes mellitus for more than one year, with fasting blood glucose levels of at least 126 mg/dl or postprandial blood glucose levels of at least 200 mg/dl. Patients with smoking habits, renal or hepatic impairment, uncontrolled hypertension, or diabetic complications were excluded from the study in order to avoid confounding influences on the outcomes and to ensure participant safety during exercise training. Before initiation of the intervention, all participants underwent baseline assessment and pre-intervention stress testing. The two groups were found to be comparable at baseline, with no statistically significant differences in age group distribution, mean age, gender distribution, height, weight, desired weight, or weight change readiness, indicating that the sample was homogeneous and suitable for comparative analysis. In the aerobic exercise group, the mean age was 53.3 years, while in the resistance exercise group it was 54.9 years and the difference was not statistically significant. Similarly, gender distribution was comparable, with females constituting the majority in both groups. The exercise intervention in both groups was structured and supervised and the intensity was standardized according to the Modified Borg Rating of Perceived Exertion scale, with participants exercising at an intensity corresponding to a rating of 4, described as somewhat hard. The aerobic exercise group performed structured aerobic training intended to improve cardiovascular endurance and glucose utilization through continuous rhythmic physical activity. The resistance exercise group underwent structured resistance training focused on major muscle groups with the aim of improving muscular strength, metabolic efficiency and insulin sensitivity. Supervision was maintained throughout the intervention period to ensure adherence to the prescribed protocol, correct execution of the exercises and safety of the participants. Outcome assessment was carried out at baseline and during follow-up at multiple time intervals, including 3 months, 6 months, 9 months and 12 months, with long-term evaluation extending up to 18 months in the trial framework. The parameters assessed included glycaemic variables such as HbA1c, fasting blood glucose and postprandial blood glucose, cardiovascular variables such as systolic and diastolic blood pressure and lipid profile parameters including HDL, LDL, total cholesterol and triglycerides. In addition to these clinical and biochemical variables, selected behavioural and self-perceived measures such as perceived blood glucose control, weight change readiness, diet knowledge and skills, diet change readiness, diet decision making, eating problems and diet barriers were also compared between the two groups over time. The collected data were analyzed statistically using appropriate methods. Baseline comparisons between the two groups were performed using chi-square tests for categorical variables and tests of mean difference for continuous variables. For repeated comparisons across time intervals, analysis of variance and Mann-Whitney tests were used where appropriate and Bonferroni post-hoc comparisons were applied to identify differences across specific time points. Ethical approval for the study was obtained from the Institutional Ethical Committee and the study was conducted in accordance with ethical standards after obtaining consent from participants.

## Results:

Table 1 presents the comparison of participants in the aerobic exercise (AE) and resistance exercise (RE) groups according to age-group distribution. In the AE group, 30.00% of subjects were in the 45-49 years' category, 26.67% were in the 50-54 years' category, 26.67% were in the 55-59 years' category and 16.67% were in the 60-67 years' category. In the RE group, 20.00% of subjects were aged 45-49 years, 23.33% were aged 50-54 years, 30.00% were aged 55-59 years and 26.67% were aged 60-67 years. Thus, both groups had participants distributed across comparable age categories, although the resistance exercise group had a slightly higher proportion of older participants in the 60-67 years' category. The chi-square analysis showed that this difference in age-group distribution between the two groups was not statistically significant (Pearson chi^2^ (3) = 1.4178, p = 0.701), indicating that the groups were comparable with respect to age composition at baseline. The mean age in the aerobic exercise group was 53.3 years with a standard deviation of 5.15, whereas in the resistance exercise group the mean age was 54.9 years with a standard deviation of 5.96. The mean difference between the groups was 1.6 years and this difference was also statistically non-significant (p = 0.2707). These findings suggest that age was well balanced between the two intervention groups before the commencement of the exercise program, thereby reducing the possibility of age acting as a confounding factor in the comparison of outcomes. Table 2 presents the gender-wise distribution of subjects in both groups. In the AE group, 20.00% were males and 80.00% were females, whereas in the RE group, 13.33% were males and 86.67% were females. The difference in gender distribution was statistically non-significant (Pearson chi2 = 0.4800, p = 0.488), indicating that the two groups were comparable with respect to sex distribution at baseline. Table 3 compares mean height, weight, desired weight and weight change readiness between the two groups. The mean height was 150.13 ± 12.49 cm in the AE group and 154.83 ± 10.05 cm in the RE group. Mean body weight was 71.03 ± 15.09 kg in the AE group and 89.2 ± 112.43 kg in the RE group. Desired weight was 56.0 ± 8.12 kg in the AE group and 55.3 ± 5.63 kg in the RE group. Weight change readiness score was 1.7 ± 0.47 in the AE group and 1.53 ± 0.51 in the RE group. None of these baseline differences were statistically significant, confirming that both groups were comparable in anthropometric variables and readiness for weight change before intervention. The study further compared the effects of Aerobic Exercise (AE) and Resistance Exercise (RE) on glycemic control parameters over a one-year follow-up period among participants. The parameters evaluated included HbA1C levels, fasting blood glucose levels, and postprandial blood glucose levels. The findings demonstrated significant improvements in both groups over time, with the Aerobic Exercise group consistently showing slightly greater reductions compared to the Resistance Exercise group. Glycated hemoglobin (HbA1C %) levels were assessed at baseline and at multiple follow-up intervals to evaluate long-term glycemic control. As presented in Table 4, the baseline HbA1C levels were comparable between the two groups, with mean values of 6.71 ± 0.59 in the Aerobic Exercise group and 6.77 ± 0.45 in the Resistance Exercise group. A gradual decline in HbA1C levels was observed in both groups throughout the study duration. After 7 days, the HbA1C values reduced slightly to 6.60 ± 0.59 in the AE group and 6.72 ± 0.46 in the RE group. Progressive reductions continued over time, reaching 6.42 ± 0.59 and 6.55 ± 0.49 after 3 months, respectively. At the end of 1 year, the Aerobic Exercise group demonstrated a mean HbA1C value of 6.19 ± 0.59, while the Resistance Exercise group showed a mean value of 6.23 ± 0.49. Statistical analysis revealed significant reductions in HbA1C levels within both groups, with p-values of 0.0050 and 0.0001, respectively, indicating that both exercise modalities contributed effectively to improved long-term glycemic control (Table 4). Fasting blood glucose levels were also evaluated to determine the effectiveness of exercise interventions on baseline glucose regulation. As shown in Table 5, both groups had similar fasting blood glucose levels at baseline, with mean values of 166.33 ± 15.41 mg/dL in the Aerobic Exercise group and 165.87 ± 12.33 mg/dL in the Resistance Exercise group. Following initiation of the exercise interventions, fasting blood glucose levels showed a steady decline in both groups. After 3 months, the fasting glucose levels decreased to 148.83 ± 14.9 mg/dL in the AE group and 151.93 ± 12.31 mg/dL in the RE group. Continued reductions were noted at subsequent follow-up intervals. By the end of one year, the Aerobic Exercise group achieved a mean fasting blood glucose level of 133.33 ± 15.06 mg/dL, whereas the Resistance Exercise group recorded a mean level of 135.47 ± 12.51 mg/dL. The observed reductions in fasting blood glucose levels were statistically highly significant in both groups (p = 0.0001), highlighting the beneficial impact of regular physical exercise on fasting glycemic regulation (Table 5). Postprandial blood glucose levels were additionally assessed to evaluate glucose metabolism following meals. According to Table 6, the baseline postprandial glucose levels were markedly elevated in both groups, with mean values of 317.47 ± 37.50 mg/dL in the Aerobic Exercise group and 322.63 ± 42.06 mg/dL in the Resistance Exercise group. A gradual decline in postprandial glucose levels was observed over the study period in both intervention groups. After 3 months, the values decreased to 288.90 ± 36.97 mg/dL in the AE group and 297.87 ± 39.96 mg/dL in the RE group. Further reductions were observed at 6 months, 9 months, and 1 year. At the final follow-up, the Aerobic Exercise group exhibited a mean postprandial glucose level of 262.90 ± 36.85 mg/dL, whereas the Resistance Exercise group demonstrated a value of 285.17 ± 43.52 mg/dL. Statistical analysis showed highly significant improvements in postprandial glucose control in both groups, with p-values of 0.0001 for the Aerobic Exercise group and 0.0017 for the Resistance Exercise group (Table 6). Overall, the findings of the study demonstrated that both Aerobic Exercise and Resistance Exercise significantly improved glycemic control over time, as evidenced by reductions in HbA1C%, fasting blood glucose, and postprandial blood glucose levels. However, the Aerobic Exercise group consistently exhibited slightly greater improvements across all measured parameters compared to the Resistance Exercise group. These findings emphasize the important role of structured physical activity in the long-term management of glycemic status and suggest that aerobic exercise may offer marginally superior benefits in improving metabolic control. The study therefore highlights the importance of incorporating regular exercise interventions into diabetes management protocols to enhance glycemic outcomes and reduce the risk of long-term complications.

## Discussion:

Diabetes mellitus is a chronic metabolic disorder characterized by persistent hyperglycaemia resulting from insulin resistance, impaired insulin secretion, or both. Exercise is universally recommended as a core component of diabetes management due to its beneficial effects on glucose metabolism, cardiovascular risk reduction and overall quality of life [9]. Aerobic exercise (AE) and resistance exercise (RE) are the two most commonly prescribed exercise modalities, yet debate persists regarding their comparative effectiveness on glycaemic parameters. The present randomized controlled trial therefore aimed to compare the long-term effects of AE and RE on glycaemic outcomes in patients with type 2 diabetes mellitus (T2DM), with special emphasis on HbA1c, fasting blood glucose (FBG) and post-prandial blood glucose (PPBG) [10]. In the present study, HbA1c levels showed a significant and progressive reduction in both the aerobic and resistance exercise groups over the one-year follow-up period. Umpierre *et al.* (2011) [11] reported that both aerobic and resistance training significantly reduced HbA1c, with supervised exercise showing superior outcomes compared to advice alone. Similarly, a meta-analysis by Jansson *et al.* (2022) [12] confirmed that resistance training produces clinically meaningful reductions in HbA1c, particularly when performed at moderate to high intensity. Despite these improvements, the present study did not demonstrate a statistically significant independent effect of exercise modality on HbA1c reduction after adjustment for age, sex and follow-up duration. Instead, two-way ANOVA revealed that time (follow-up period) was the strongest determinant of HbA1c change. This observation aligns with findings by Boulé *et al.* [13] who reported that improvements in HbA1c are largely dependent on sustained exercise adherence rather than the specific type of exercise performed. This highlights the importance of long-term behavioral compliance rather than short-term modality selection. In contrast, contradictory evidence exists suggesting superiority of resistance exercise over aerobic exercise for HbA1c reduction. Tokmakidis *et al.* [14] demonstrated that resistance training led to greater reductions in HbA1c compared to aerobic training over a 16-week period. The present study demonstrated a statistically significant reduction in fasting blood glucose levels in both AE and RE groups across all follow-up points. A systematic review by Snowling and Hopkins [15] reported significant reductions in fasting glucose following both aerobic and resistance training interventions in adults with T2DM. However, when regression analysis was applied in the present study, fasting blood glucose did not remain a significant independent predictor after adjustment, indicating that reductions in FBG were largely time-dependent. This finding is consistent with observations by Sigal *et al.* [16] who reported that while fasting glucose improves with exercise, the magnitude of improvement diminishes after controlling for baseline glycaemia and intervention duration. Contradictory evidence has been reported in several PubMed-indexed trials suggesting a preferential benefit of aerobic exercise on fasting glucose. DiPietro [17] found that moderate-intensity aerobic exercise produced greater reductions in fasting glucose compared to resistance training, particularly in older adults with impaired glucose tolerance. Similarly, Church *et al.* [18] and Amaravadi *et al.*. [19] observed that combined aerobic and resistance training was more effective than resistance training alone in lowering fasting glucose. These differences may be explained by the greater influence of aerobic exercise on hepatic glucose metabolism during prolonged energy expenditure. The present study therefore makes an important contribution by demonstrating that aerobic exercise provides superior clinical improvement in PPBG, while resistance exercise also exerts a statistically significant effect, highlighting the complementary roles of both modalities. Among all glycaemic parameters assessed, PPBG emerged as the most sensitive discriminator between exercise types, addressing a major gap in comparative exercise research.

## Conclusion:

Structured aerobic and resistance training both produced significant improvements in glycaemic control, lipid profile and cardiovascular parameters in patients with type 2 diabetes mellitus. Resistance training showed greater early-phase benefits in behavioral and metabolic outcomes, although long-term effects were comparable between both modalities. Thus, both exercise strategies can be effectively incorporated into diabetes management, with selection tailored to patient-specific goals and adherence potential.

## Figures and Tables

**Table 1:** Comparison between two groups according to age-group (years) of subjects

**Age-Group (years)**	**Aerobic Exercise (AE)**		**Resistance Exercise (RE)**	
	No.	%	No.	%
45 - 49	9	30	6	20
50 - 54	8	26.67	7	23.33
55 - 59	8	26.67	9	30
60 - 67	5	16.67	8	26.67
Total	30	100	30	100
Pearson chi2(3) = 1.4178 P value = 0.701				
Mean	53.3		54.9	
SD	5.15		5.96	
Difference	1.6			
P value	0.2707			

**Table 2:** Comparison between two groups according to gender of subject

**Gender**	**Aerobic Exercise (AE)**		**Resistance Exercise (RE)**	
	No.	%	No.	%
Male	6	20	4	13.33
Female	24	80	26	86.67
Total	30	100	30	100
Pearson chi^2^(1) = 0.4800 P value = 0.488 (Non-Significant)				

**Table 3:** Comparison of mean height (cm.), weight (kg.), desired weight (kg.) and weight change readiness, in two groups

**Variable**	**Aerobic Exercise**		**Resistance Exercise**		**Mean**	**P**
	(AE)		(RE)		diff.	value
	Mean	SD	Mean	SD		
Height	150.13	12.49	154.83	10.05	-4.7	0.1138
(cm.)						
Weight	71.03	15.09	89.2	112.43	-18.17	0.384
(Kg.)						
Desired weight	56	8.12	55.3	5.63	0.7	0.6995
(Kg.)						
Weight change readiness	1.7	0.47	1.53	0.51	0.17	0.1904

**Table 4:** comparison of mean HbA1C% (Post Test) between two groups

		**Summary of Mean HbA1C % (Post Test) between two groups**		
Time Interval	Aerobic Exercise (AE)		Resistance Exercise (RE)	
	Mean	SD	Mean	SD
Baseline	6.71	0.59	6.77	0.45
After 7 days	6.6	0.59	6.72	0.46
After 3 months	6.42	0.59	6.55	0.49
After 6 months	6.34	0.59	6.45	0.55
After 9 months	6.26	0.59	6.33	0.49
After 1 year	6.19	0.59	6.23	0.49
P value	0.0050 (S)		0.0001 (S)	

**Table 5:** comparison of Mean Blood Glucose (Fasting) between two groups

		**Summary of Mean Blood Glucose (Fasting) between two groups**		
Time Interval	Aerobic Exercise (AE)		Resistance Exercise (RE)	
	Mean	SD	Mean	SD
Baseline	166.33	15.41	165.87	12.33
After 7 days	162.37	15.39	162.67	12.25
After 3 months	148.83	14.9	151.93	12.31
After 6 months	144.37	14.74	147.1	12.28
After 9 months	139.87	14.79	142.77	12.24
After 1 year	133.33	15.06	135.47	12.51
P value	0.0001 (S)		0.0001 (S)	

**Table 6:** comparison of Mean Prandial Blood Glucose (Post-test) between two groups

		**Summary of Mean Prandial Blood Glucose (Post-test) between two groups**		
Time Interval	Aerobic Exercise (AE)		Resistance Exercise (RE)	
	Mean	SD	Mean	SD
Baseline	317.47	37.5	322.63	42.06
After 7 days	311.7	35.03	316.07	41.24
After 3 months	288.9	36.97	297.87	39.96
After 6 months	275.4	38.12	296.97	44.23
After 9 months	269.77	36.74	286.7	39.35
After 1 year	262.9	36.85	285.17	43.52
P value	0.0001 (S)		0.0017 (S)	
